# Reopening International Borders without Quarantine: Contact Tracing Integrated Policy against COVID-19

**DOI:** 10.3390/ijerph18147494

**Published:** 2021-07-14

**Authors:** Zidong Yu, Xiaolin Zhu, Xintao Liu, Tao Wei, Hsiang-Yu Yuan, Yang Xu, Rui Zhu, Huan He, Hui Wang, Man Sing Wong, Peng Jia, Song Guo, Wenzhong Shi, Wu Chen

**Affiliations:** 1 Department of Land Surveying and Geo-Informatics, Hong Kong Polytechnic University, Hong Kong, China; zidong.yu@connect.polyu.hk (Z.Y.); xiaolin.zhu@polyu.edu.hk (X.Z.); xintao.liu@polyu.edu.hk (X.L.); yang.ls.xu@polyu.edu.hk (Y.X.); felix.zhu@polyu.edu.hk (R.Z.); ls.charles@polyu.edu.hk (M.S.W.); peng.jia@polyu.edu.hk (P.J.); john.wz.shi@polyu.edu.hk (W.S.); 2 School of Psychology, Shenzhen University, Shenzhen 518060, China; tao.wei@szu.edu.cn; 3 Department of Biomedical Sciences, Jockey Club College of Veterinary Medicine and Life Sciences, City University of Hong Kong, Hong Kong, China; sean.yuan@cityu.edu.hk; 4 Centre for Applied One Health Research and Policy Advice, City University of Hong Kong, Hong Kong, China; 5 School of Public Administration, Southwestern University of Finance and Economics, Chengdu 611130, China; hehuan@swufe.edu.cn; 6 Institute for Modeling Collaboration and Innovation, University of Idaho, Moscow, ID 83844, USA; huiwang@uidaho.edu; 7 Department of Computing, Hong Kong Polytechnic University, Hong Kong, China; cssongguo@comp.polyu.edu.hk

**Keywords:** COVID-19, border reopening, digital contact tracing, international travel

## Abstract

With the COVID-19 vaccination widely implemented in most countries, propelled by the need to revive the tourism economy, there is a growing prospect for relieving the social distancing regulation and reopening borders in tourism-oriented countries and regions. This need incentivizes stakeholders to develop border control strategies that fully evaluate health risks if mandatory quarantines are lifted. In this study, we have employed a computational approach to investigate the contact tracing integrated policy in different border-reopening scenarios in Hong Kong, China. Explicitly, by reconstructing the COVID-19 transmission from historical data, specific scenarios with joint effects of digital contact tracing and other concurrent measures (i.e., controlling arrival population and community nonpharmacological interventions) are applied to forecast the future development of the pandemic. Built on a modified SEIR epidemic model with a 30% vaccination coverage, the results suggest that scenarios with digital contact tracing and quick isolation intervention can reduce the infectious population by 92.11% compared to those without contact tracing. By further restricting the inbound population with a 10,000 daily quota and applying moderate-to-strong community nonpharmacological interventions (NPIs), the average daily confirmed cases in the forecast period of 60 days can be well controlled at around 9 per day (95% CI: 7–12). Two main policy recommendations are drawn from the study. First, digital contact tracing would be an effective countermeasure for reducing local virus spread, especially when it is applied along with a moderate level of vaccination coverage. Second, implementing a daily quota on inbound travelers and restrictive community NPIs would further keep the local infection under control. This study offers scientific evidence and prospective guidance for developing and instituting plans to lift mandatory border control policies in preparing for the global economic recovery.

## 1. Introduction

According to the World Tourism Organization (UNWTO), 2020 was documented as the worst year for international tourism with an unprecedented recession of 74% regarding international arrivals compared to previous year [1]. This economic downturn was caused by the global spread of the coronavirus disease 2019 (COVID-19), where many countries closed their borders to contain the virus spread resulted from international travel and trade. These border control strategies had a lasting impact on countries and regions relying on the tourism economy, such as France, Thailand, and Hong Kong, China [2]. Further, the shortage of cross-border labor was predisposed to restrict the production capacity in certain industries, such as outsourced agriculture [3]. As phases of COVID-19 vaccination are rolling out and new cases are declining in most world regions, growing discussion arises about when and how to lift international travel bans and reopen borders with an eye to reviving the economy from the global calamity.

To minimize the risk for epidemic resurgence, some countries have partially reopened their borders by implementing strict quarantine rules on all incoming travelers. For example, Canada imposed mandatory nucleate tests as well as a 14-day quarantine policy (i.e., 3-day institutional quarantine and 11-day self-quarantine) on all incoming travelers. In Hong Kong, all incoming travelers were required to undergo a 14-day or 21-day mandatory quarantine at designated institutions, where the quarantine period was determined on the risk level of their country of origin. More importantly, the mandatory quarantine helped public health officials to isolate cases in the incubation period before the symptom onset. Although these strict containment measures were proven to be effective in reducing imported cases and their potential secondary local transmission, they were criticized for posing considerable obstacles for human movement and international tourism [4,5,6]. As a result, stakeholders were pressed to reevaluate the health risk associated with the possibility of reopening borders without an elongated period of quarantine to offset the socioeconomic loss. Eventually, it is expected that the reopening policy will achieve an intricate balance between acceptable levels of health risks and steady socioeconomic recovery.

Nonpharmaceutical interventions (NPIs), which can be considered as mitigation strategies apart from medical treatments, have been implemented in most countries to mitigate the spread of the disease. NPIs for domestic containment can be broadly categorized into two strategies, namely, social distancing measures and contact tracing. Social distancing measures focused on reducing the risk of infection through personal protection (i.e., facemask rules) or community interventions [7]. Community-level social distancing measures, such as school arrangement, work-from-home arrangements, and limited opening of nonessential businesses, were effective to lower the intensity of interpersonal interactions and thus alleviate the health impact before the vaccine became widely available [8,9,10].

Furthermore, contact tracing is a promising way to control the spread of infectious cases. It was demonstrated in clinical research and simulation results that contact tracing was highly effective in identifying the etiology of transmission and helping maintain low infection levels [11,12,13]. Given that a local spread can quickly induce a spike of new cases, conventional contact tracing methods, such as the travel diary survey, were characterized by a delay between confirming a new case and locating the patient’s close contacts [14]. In contrast, digital contact tracing enabled by location-based devices (e.g., mobile phones and tracking wristbands) makes epidemiological investigation highly efficient and the follow-up notification process in near real-time upon the confirmation of new cases [15].

With the aim to reopen international borders, some researchers have articulated the potential effects of the imported virus carriers that may raise secondary local transmission in communities and relieving NPIs [6,16]. However, these recommendations were mostly based on mandatory quarantines, and there has been limited scientific exploration to quantify the risk of COVID-19 if borders are reopened and mandatory quarantines are lifted. This oversight could be problematic since the pathway towards rehabilitating the travel normalcy must include a thorough understanding of the health risk if the quarantine process is to be circumvented for international travel.

To this end, there is an urgent need to formulate a sustainable strategy for reopening international borders by relieving the quarantine requirements while simultaneously applying digital contact tracing. In this paper, we propose a framework to integrate the importation risk induced by potential virus carriers into a mechanistic epidemic model in a case study of Hong Kong. We aim to answer two important research questions: (1) To what extent can digital contact tracing help contain the local infection compared to scenarios without contact tracing? (2) To what extent can the containment measures be improved, such as combining digital contact tracing with the inbound quota and community NPIs?

## 2. Methods

### 2.1. Data

This study employs the daily reported COVID-19 cases from 27 January 2020 up to 5 May 2021 provided by the Centre for Health Protection (CHP) of the Department of Health in Hong Kong, which released the COVID-19 data and made it freely accessible on the government’s open-data platform (https://data.gov.hk/en-data/dataset/hk-dh-chpsebcddr-novel-infectious-agent, accessed on 10 May 2021). All recorded cases of infection were laboratory- and clinical-confirmed via nasopharyngeal swab and PCR with reverse transcription (RT-PCR) [17]. The data depict the details of the confirmed cases that are related to individual patients, comprising case number, reported and onset dates, case classification, and other demographic information. Such empirical evidence in Hong Kong can provide an insight into studying the process of case spread.

In response to the imported COVID-19 cases, the government fully implemented a mandatory quarantine scheme on 25 December 2020 on all inbound visitors, aiming to cut off the contact between infectious cases and local communities. We assume that the imported cases are not attributed to the local spread because of the screening and mandatory quarantines imposed on all incoming travelers since December 2020. To estimate the local spread of COVID-19 in Hong Kong, we thus consider local cases and cases epidemiologically linked to local cases from 25 December 2020 through 5 May 2021.

### 2.2. Estimation of SEIR Model Parameter

A classic stochastic SEIR (susceptible–exposed–infectious–recovered) model is proposed to estimate the course of the epidemic development by fitting the observed cumulative daily reported cases. As suggested by previous literature, the SEIR model has been extensively employed to simulate and analyze the dynamics of COVID-19 pandemic in the context of Hong Kong [18,19,20]. As shown below, the total population (*N*) is partitioned into four compartments, which are susceptible (*S*), exposed (*E*), infectious (*I*), and removed/recovered (*R*). Among these compartments, removed/recovered (*R*) is further subdivided into population removed from infectiousness (*I*) and vaccinated population from susceptible (*S*) during the period of vaccination.

N=S+E+I+R

For each time epoch (day), four ordinary differential equations are performed to express the dynamic progression (daily changes) between different epidemiological compartments in the SEIR model, as follows:

$$\frac{dS}{dt}=-\beta_{d}\frac{SI}{N}-\mu \delta S$$

$$\frac{dE}{dt}=\beta_{d}\frac{SI}{N}-\sigma E$$

$$\frac{dS}{dt}=\sigma E-\gamma S$$

$$\frac{dR}{dt}=\gamma S+\mu \delta S$$

The number of new infections on a daily basis is calculated based on the total contacts between susceptible and infected individuals, of which the transmission rate β varies across different regions and countries. The initial β is estimated using the basic reproduction number of observed cumulative cases, divided by the infectious period from symptoms onset to the first medical treatment. Here, the incubation period 1σ and infectious period 1γ are set to be 5.5 days (95% CI: 4.5–5.8) and 7.1 days (95% CI: 2.5–11.6), respectively [21,22].

Two modifications are proposed in our model fitting to adapt the local disease etiology. One modification is to consider six main interventions released in the past few months in Hong Kong, including school arrangement, suspension of large events, public facility arrangement, restricting social gathering, using the voluntary check-in system, and compulsory testing, according to a series of NPIs implemented in communities to reduce contacts between the susceptible and potentially infected individuals (Table 1). The strictness of a specific NPI is represented by using a multiplier to amplify its base value, for example, the reduction effectiveness of school arrangement $\alpha_{school}$ at strict and moderate levels is assumed to be three times and two times larger than that at the mild level, respectively. As these interventions have been updated over time (Supplemental Figure S1), transmission rate *β* is then multiplied by the coefficient of various implemented NPIs $\alpha_{NPIs}$, for recapitulating the trend of local infections more precisely, as follows:

$$\beta_{d}=\beta \times \alpha_{NPIs}$$

$$\alpha_{NPIs}=\alpha_{school}\times \alpha_{event}\times \alpha_{facility}\times \alpha_{gathering}\times \alpha_{check-in}\times \alpha_{testing}$$

Another modification is the territory-wide COVID-19 vaccination program for all Hong Kong residents since 27 February 2021, which is proposed as an essential public health barrier against COVID-19. According to the cumulative vaccinated population, the Levenberg–Marquardt algorithm is employed to estimate the daily vaccination coverage δ of the susceptible population before 5 May 2021 [23]. We further consider the probability of inducing an immune response regarding the vaccinated population, since some individuals are unable to be vaccinated for vaccine breakthrough infections [24]. Therefore, the effectiveness μ of the vaccines adopted in Hong Kong is deployed to specify the vaccinated population that would actually acquire immunity [25,26]. The vaccinated population would not infect or be infected by infectious individuals.

A set of designated parameters $\theta =\left\{\beta ,\sigma ,\gamma ,\;\alpha_{NPIs}\;\right\}$ are subject to statistical inference, including infectious rate, incubation rate, recovery rate, and the coefficients of various implemented NPI. We estimate these parameters by determining the best fitting between the simulated and the confirmed daily cumulative cases in Hong Kong. The results from parameter fitting process would be a posterior distribution of designated parameters θ. The statistical inference is calibrated within a Bayesian optimization framework prior to the Markov chain Monte Carlo simulation.

### 2.3. Simulation of the Effectiveness of Digital Contact Tracing

Because of the potential virus carriers during the border-reopening phase, it is expected that the COVID-19 resurgence in Hong Kong is highly possible. Hypothetical scenarios are proposed to understand whether digital contact tracing can help to contain the importation risks when only a proportion of local residents are vaccinated. We assume 152,473 people per day will enter this city based on the historical visitor data in 2019 [27]. The model imposes digital contact tracing on all inbound travelers during their entire stay in Hong Kong. Specifically, susceptible population (S) who closely contact with infectious travelers (I) will be immediately quarantined or isolated, becoming removed population (R) instead of being exposed or infectious population existing in the community. The schematic to explain this rationale is shown in Supplemental Figure S2.

Since Hong Kong has imposed extremely strict screening measures including mandatory pre-departure and arrival tests on all inbound travelers, we assume that only susceptible $T_{susceptible}$ and exposed travelers $T_{exposed}$ can enter this city. Hence, the overall importation risk ε is represented as the percentage of inbound travelers exposed to the virus upon total arrival, which is based on the total number of imported cases and its ratio to the historical arrival population retrieved from Immigration Department during our model fitting period. The daily inbound population exposed to the virus is represented as a sum of a random sample drawn from a Poisson distribution, as follows:

$$\frac{dS}{dt}=T_{susceptible}-\beta_{d}\frac{SI}{N}-\mu \delta S$$

$$\frac{dE}{dt}=\beta_{d}\frac{SI}{N}+T_{exposed}-\sigma E$$

$$T_{exposed}=\varepsilon \times \left(T_{susceptible}+T_{exposed}\right)$$

As suggested by the historical arrivals in 2019 with an average stay of 3.3 nights for visitors, we further modify our model by deducting a daily outflow population from our model based on samples from a Poisson distribution (λ = 3.3) [27]. As most people tend to visit a city for a few days, such a setting can simulate a more realistic travel behavior. The total population exiting Hong Kong $EX_{d}$ on day d is represented as follows:

$$EX_{d}\sim \;\frac{\lambda^{d}{ℯ}^{\lambda }}{d!}\left(T_{susceptible}+T_{exposed}\right)$$

### 2.4. Incorporating Joint Inflow Population Control and Community NPIs

Although digital contact tracing can help contain the local infection, its implementation cannot ensure a promising health outcome in the most realistic or the worst-case scenario (30% vaccination coverage). Considering that only a small susceptible population is vaccinated, in addition to contact tracing, it is imperative to estimate the joint effects of controlling arrival population and implementing community NPIs [28]. In this regard, a set of scenarios combining the daily quota and the community NPIs are proposed.

With respect to the daily quota, three different border-reopening policies are simulated. Compared to the baseline scenario with a daily arrival population of 152,473, two other quotas are set: 76,236 per day (50% of historical arrivals) and 10,000 per day, respectively. Limiting the daily inbound population can control the potential importation of virus carriers, thus reducing the number of active exposures in the SEIR model. Simultaneously, community NPIs are also implemented during the border-reopening phase, with the aim to lower the transmission rate between the infectious and susceptible populations. The proposed community NPIs are on a moderate-to-strict level with school arrangement, suspension of large events, public facility arrangement, restricting social gathering, using the voluntary check-in system, and compulsory testing (Supplemental Table S1). NPIs used in our simulations encourage people to practice social and physical distancing. Compared to mandatory contact tracing and restricting arrival population, community NPIs have severe socioeconomic adversities in people’s daily life. Thus, the NPIs adopted in this study exclude the most restrictive scenarios, such as regional lockdown and school closures.

In our simulation, all close contacts are ideally (100%) self-isolated or mandatory quarantined without secondary infections. However, we recognize that it is impossible to achieve such ideal efficiency (100% close contacts can be located and treated), as such, we additionally provide a more realistic simulation setting in which only 90% of close contacts can be found. In other words, 10% of close contacts are released into local communities as the exposed and thus infectious population.

## 3. Results

### 3.1. Deriving Parameters from Empirical Data Using a Modified SEIR Model

**Figure 1:**
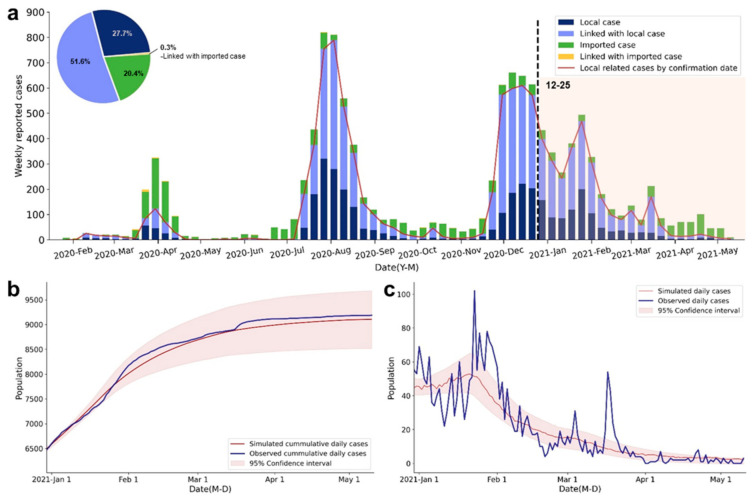
Reported cases and fitted curve of the SEIR model in Hong Kong. (**a**) The epidemic curve by weekly reported cases that belong to different case categories: local cases, cases linked to local cases, imported cases, and cases linked to imported cases. The dashed line and color band indicate the period of observation for model fitting. (**b**). Comparison of the observed and simulated cumulative daily cases. The color shaded area represents the 95% CI of the simulated cases. (**c**). Comparison of the observed and simulated daily cases. The color shaded area represents the 95% CI of the simulated cases.

To estimate the course of local infections, we have extracted confirmed cases in Hong Kong, which were categorized as either local cases or cases linked to local cases from 25 December 2020 through 5 May 2021 (Figure 1a). The classic process-based susceptible–exposed–infectious–removed (SEIR) model is modified to account for local factors affecting the transmission. These factors include the community NPIs and the regional COVID-19 vaccination program.

The modeling results show a relatively high level of correspondence with observed cumulative cases, confirming that our modification of the classical SEIR model can well preserve the dynamic course of local infections (R^2^ = 0.99; RMSE = 129.04; Figure 1b). The simulated daily new cases, derived from the fitted model, are compared with observed daily cases (Figure 1c). However, the predictive results are less precise for January and from 12 March to14 March, of which some cases are beyond the 95% confidence intervals (CI). Such deviations could be attributed to the stochastic nature of the local outbreaks, such as the superspreading events (SSEs) in a low-prevalence context. SSEs can dramatically increase the confirmed incidences in a short period [29]. Explicitly, one explanation of the inconsistency during mid-to-late January is the explosive growth of reported cases due to the massive untraceable SSEs in Yau Ma Tei and Jordan districts. In addition, our prediction underestimates the reported cases dated from 12 March to14 March, which are mostly associated with the outbreak of the URSUS Fitness cluster. Despite these SSEs, the modified SEIR model has a satisfactory capacity to elucidate the course of local infections via a robust simulation process.

The fitted parameters are estimated from our model by relating to the daily transmission rate (Supplemental Figure S3). Among these NPIs, compulsory testing is estimated to have the largest alleviating effect, reducing the disease transmissivity by 48.69% (95% CI: 30.29–51.93%) The results provide evidence that the interventions in the past few months were largely effective in reducing the transmission rate.

### 3.2. Simulating the Effects of Contact Tracing on Reopening Borders

In this section, we present our simulation results for estimating the difference between two designated baseline scenarios during the potential border-reopening phase: one with digital contact tracing and the other without digital contact tracing (i.e., conventional SEIR model). In addition, due to the vaccination program in Hong Kong, we simulate three base scenarios with different vaccination coverages (30%, 50%, and 70%) to understand whether digital contact tracing can substitute the mandatory quarantine while minimizing the importation risk of COVID-19.

**Figure 2:**
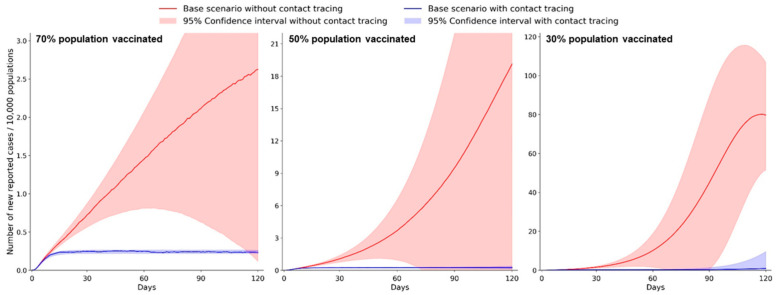
Estimated effectiveness of digital contact tracing in containing the importation risk for reopening borders during the 120-day simulation period. The daily reported cases are estimated under three different vaccination coverages (30%, 50%, and 70%). The color shaded area shows the 95% CI of estimated daily reported cases.

The effectiveness of using digital contact tracing is estimated by assuming the full quarantine (100%) of close contacts upon case identification (Figure 2 and Supplemental Figure S4). A large difference regarding the simulated daily cases from active infectious patients can be clearly observed between all baseline scenarios with and without contact tracing. In particular, 68.23%, 91.07%, and 97.24% of the infectious population are predicted to be isolated under the scenarios with 70%, 50%, and 30% vaccination coverage, respectively. Another finding is that a higher vaccination coverage of the residents can more effectively contain the virus spread by reducing the susceptible population. The optimal scenario can be concluded from the results, where applying digital contact tracing on all inbound travelers and a 70% vaccination coverage rate can largely flatten the epi curve and maintain the infections on a relatively low level. In this optimal scenario, we estimate the daily reported cases to be 188 (95% CI: 175–210) and the cumulative infection cases to be 19,163 (95% CI: 17,393–21,761) on the 60th day of reopening the border. It is noted that the number of cumulative infections includes reported cases identified from infectious population in communities and close contacts that are exposed to inbound infectious travelers during their subsequent mandatory quarantine. However, as of 26 May 2021 or 88 days since the initiation of the vaccination program, only 934,399 (12.5% of the total population) received complete vaccination, meaning that the actual vaccinated population was likely to be far below the theoretical threshold for reaching herd immunity [30]. As such, we simulate the effectiveness of digital contact tracing under the most pessimistic but more realistic scenario with 30% vaccination coverage. In such settings, the daily reported cases are predicted to be 218 (95% CI: 110–268), and the cumulative infection cases are 26,530 (95% CI: 22,103–31,602) on the 60th day of reopening the border.

### 3.3. Joint Effects of Controlling Arrival Population and Community NPIs

As shown above, the application of digital contact tracing can effectively contain the importation risk of COVID-19; however, it is less likely for public health officials to well control the transmission in the worst-case scenario (30% vaccination coverage). Hence, it is imperative to estimate the joint effects of controlling the inbound population and community NPIs. Specifically, we factor in the joint effects of applying a daily quota on inbound travelers and various degrees of community NPIs. Compared to the historical population flow (152,473 per day) in the baseline scenarios, two daily quotas are set: 76,236 (50% of historical arrivals) and 10,000. Additionally, moderate-to-strong community NPIs are simultaneously applied, including the school arrangement, suspension of large events, public facility arrangement, restricting social gathering, using voluntary check-in system, and compulsory testing (Supplemental Figure S5).

**Figure 3:**
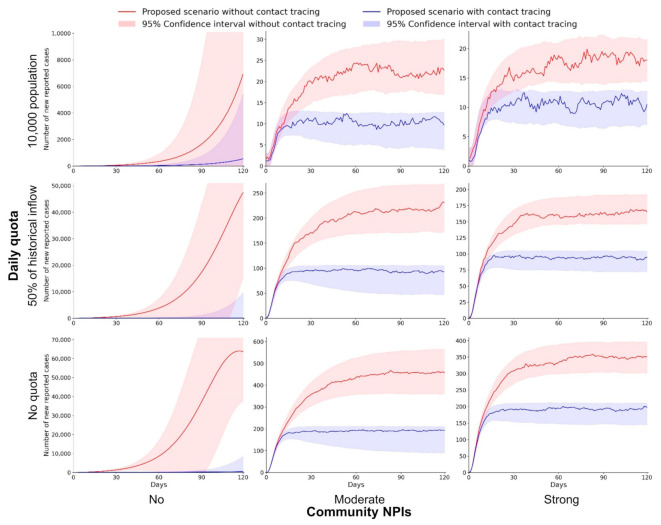
Implementing a daily quota for inbound travelers and community NPIs to contain the importation risk during the 120-day simulation period. The number of daily reported cases is estimated under scenarios with different daily quotas (no quota, 50% of historical inflow, and 10,000 population) and community NPIs (no, moderate, and strong). The color shaded areas are the 95% CIs of estimated daily reported cases.

As reported in our simulation results, the joint effects of controlling for inbound travelers and applying community NPIs are predicted to further reduce the number of daily infections compared to the scenario with contact tracing only (Figure 3 and Table 2). The alleviating effects are estimated to be more substantial with a limited daily quota and more restrictive community NPIs. Without implementing any community NPIs, the cumulative reported cases during the first 60 days after reopening the border are predicted to be 5008 (95% CI: 3859–7105) and 978 (95% CI: 151–2069) under moderate and strict border control policies, respectively. Similarly, when there is no daily quota, our simulation estimates that the cumulative reported cases are 9992 (95% CI: 9213–11,150) and 10,061 (95% CI: 9168–11,119) under moderate and strong NPIs, respectively. Ideally, applying a 10,000 daily quota and moderate-to-strong community NPIs jointly can limit the cumulative reported cases to 549 (95% CI: 440–697), where the number of daily reported cases is 9 (95% CI: 8–13) on the 60th day of reopening the border.

In practical terms, digital contact tracing and follow-up intervention cannot be 100% successful for isolating infectious individuals. Hence, we further assume practical scenarios with a success rate of 90% for isolation (Supplemental Table S2 and Supplemental Figure S6). Our simulation estimates that digital contact tracing with a 10,000 daily quota and moderate-to-strong community NPIs can still ensure the infections to be maintained on a low level, in which cumulative reported cases are limited to 634 (95% CI: 457–716) during the first 60 days of reopening the border. Overall, the results suggest that for cities with low vaccination coverage, restricting inbound travelers, applying community NPIs, and implementing digital contact tracing jointly can effectively contain the importation risk of COVID-19 while reopening borders.

## 4. Discussion

Concerning the importation risk of COVID-19, existing studies have articulated strategies based on mandatory quarantines. Nevertheless, a lengthy quarantine could be unbearable for inbound travelers and can pose obstacles to the revival of the tourism economy. Using location-based devices, such as tracking wristbands, to track the close contacts of infectious patients has been proven successful in containing the virus spread [31,32]. Therefore, a simulation study is proposed to evaluate the effectiveness of digital contact tracing under different vaccination scenarios without mandatory quarantines. Key findings and contributions are discussed as follows.

By reconstructing the COVID-19 transmission from historical data, we confirm that digital contact tracing can effectively help to contain the importation risk of COVID-19 by flattening the epi curve. In the most realistic scenario with only a 30% vaccination rate, the predicted number of daily reported cases for the first 60th days after reopening the border is 174 (95% CI: 146–207), which is a significant reduction of 92.11% (95% CI: 69.90–96.02%) compared to the setting without contact tracing. This finding provides evidence that digital contact tracing and the follow-up isolation process upon case confirmation can prevent the virus from further spreading and can thus reduce the transmissibility of the disease [7,33]. Although digital contact tracing is effective, additional containment measures are still needed to curb the pandemic. One unique feature of our model is to simulate the short-term stay of inbound travelers if the mandatory quarantine is relieved, which has been less mentioned in the discussion about reopening borders [16,34]. This feature can be further applied to modeling the outflow of exposed travelers before their symptom onset in another country. This international application is highly relevant to studies of the importation risk around the globe because of the increasing international trend, travel, and migration when more borders are open.

Concurrent with digital contact tracing, restricting international travel and implementing community NPIs are recommended measures to contain the local infection. Explicitly, applying a daily quota on travelers can limit the likelihood of virus carriers entering the city, while community NPIs can further reduce the virus transmissibility. It is noteworthy that we simulate the least restrictive but sustainable criteria for guiding the reopening policy, where extreme containment measures, such as school closures, are not considered [35]. We also simulated under the optimistic scenario with digital contact tracing, a daily quota on inbound travelers, and community NPIs jointly in effect, forecasting the daily increase in a 60-day period would be around nine cases per day (95% CI: 7–12); without contact tracing, this would be around 14 cases per day (95% CI: 12–17). However, as stressed by other studies, it is intractable to implement contact tracing with instantaneous isolation of infectious patients [36,37]. In this regard, the effectiveness of contact tracing in the optimistic scenario is highly reliant upon the response capacity of the local public health department. More significantly, while contract tracing relieves the workload and resources that could be otherwise expended on mandatory quarantines, it is expected that the potential surge of disease outbreaks can become a prime concern when there is no barrier to hold imported cases.

Nevertheless, several limitations exist in our proposed framework and the designed scenarios. First, our simulation model is built on the epidemiological parameters derived from reported cases in Hong Kong. Based on recent COVID-19 studies and results of the Universal Community Testing Program, we hypothesize that all of the infectious patients were identified, diagnosed, and reported by the health surveillance system after the third wave of outbreak in Hong Kong [38,39,40]. In other words, asymptomatic patients, namely, hidden COVID-19 carriers, are assumed not to exist in our model. Nevertheless, this assumption may not be applied to other countries and regions due to the heterogeneity in case diagnosis and surveillance, where the actual infections are estimated to be much greater than the reported cases, for example, 6–24 times higher in the United States [41,42]. Second, real-time NPIs in the past few months are recorded and simulated in our model to understand their effectiveness. However, preventive behaviors on the individual level (e.g., human mobility and face covering), which dictate the likelihood of infection, are not considered in our simulation [43,44]. Thus, future extensions of the model should emphasize these behavioral factors to better estimate virus transmissibility. Third, our model can simulate the epi curve over a short period (e.g., two months) but is unable to predict results over the long term because of the many uncertainties arising from the epidemic development, such as the variants of the virus and the efficacy of vaccination [45,46]. These changing situations can be accommodated by learning and updating epidemiological parameters as the simulation progresses on a daily basis. Finally, it should be noted that there are ethical issues in the usage of digital contact tracing. Its wide implementation must be grounded on public trust and health policy about privacy protection [36]. These considerations will eventually help the public weigh the health benefits of digital contact tracing against the potential privacy issues.

## 5. Conclusions

In this research, we investigate the importation risk of COVID-19 in scenarios of reopening borders. This endeavor is an essential step to recover the economy in the post-pandemic phase. Two policy-relevant recommendations are drawn from our simulations. First, our results support that digital contact tracing is an effective countermeasure to limit case reproduction, especially when the imported case can be quickly identified and isolated. Second, restricting inbound travelers and implementing community NPIs jointly can ensure the local infection to be under control. Combined together, digital contact tracing integrated policies are illustrated and suggested as regards to lifting mandatory quarantine rules in preparing for the global economic recovery. A practical insight into sustainable border-reopening policies is provided by this study and can be considered by other countries and regions.

## Figures and Tables

**Table 1:** Description of nonpharmaceutical interventions (NPIs) implemented in Hong Kong from December 2020 to January 2021.

NPI	Level	Description
School arrangement	Strict	Suspension of face-to-face classes for all schools
	Moderate	Face-to-face classes with the number of students capped at one-third of the total number of students.
	Mild	Face-to-face classes with the number of students capped at one-third of the total number of students.
Suspension of large events	Strict	Large-scale events are canceled to reduce public risk
	Moderate	Large-scale events are partially opened
Public facility arrangement	Strict	Public facilities are temporarily closed (cultural, recreation, and sports activities)
	Moderate	Public facilities are partially opened
	Mild	Public facilities are opened with restrictions
Restricting social gathering	Strict	No more than two persons are allowed to gather within any public space
	Moderate	No more than four persons are allowed to gather within any public space
Voluntary check-in system		A mobile app allowing users to record their whereabouts and get exposure notification.
Compulsory testing		Restricted districts are delineated based on confirmed case number with a “restriction-testing declaration” to require all residents for a virus testing.

**Table 2:** Implementing a daily quota for inbound travelers and community NPIs with contact tracing (100% successful for individuals) in containing importation risk for reopening borders.

Daily Quota	Community NPIs	Cumulative Cases during the First 60 Days (95% CI)	Cumulative Cases during the First 60 Days (95% CI)	Reduction Rate of Cases (%)
		No Contact Tracing	Contact Tracing	
No	No	132,243 (41,353–220,293)	10,429 (8763–12,447)	92.11
	Moderate	18,362 (15,678–21,544)	9992 (9213–11,150)	45.58
	Strong	15,568 (13,790–17,658)	10,061 (9168–11,119)	35.37
50% of historical inflow	No	60,598 (18,690–98,721)	5008 (3859–7105)	91.73
	Moderate	8994 (7521—10,446)	5057 (4490–5592)	43.77
	Strong	7599 (6706–8590)	5039 (4520–5549)	33.68
10,000	No	5618 (1063–11,676)	978 (151–2069)	82.59
	Moderate	995 (753–1222)	562 (437–699)	43.51
	Strong	802 (654–1006)	549 (440–697)	31.54

Note: The reduction rate refers to the percentage of cases that could be reduced if the digital contact tracing is applied, compared with the identical scenario without contact tracing.

## Data Availability

Publicly available datasets were analyzed in this study. This data can be found here: https://data.gov.hk/en-data/dataset/hk-dh-chpsebcddr-novel-infectious-agent, accessed on 10 May 2021.

## Supplementary Materials

The following are available online at https://www.mdpi.com/article/10.3390/ijerph18147494/s1, Figure S1: Gantt chart for the implementation period of NPIs. Different colors represent levels of strictness in terms of the implemented NPIs, Figure S2, Schematic to explain this rationale how digital contact tracing help to contain the importation risks. In this illustration, we assume that close contacts exposed to the inflow infectious individual are ideally (100%) self-isolated or mandatory quarantined, Figure S3, Individual NPI effectiveness in reducing disease transmissivity deriving from the modified model fitting with observed daily reported cases. Results are shown using a violin plot and the box plot inside each violin display median and interquartile range, Figure S4, Estimated cumulative daily cases under three different vaccination coverage. The color shaded area shows the 95% CI of estimated cumulative cases regarding corresponding scenarios. Solid and dotted lines represent the scenarios with and without contact tracing, respectively. Different colors represent scenarios with 70%, 50%, and 30% vaccination coverage, Figure S5, Combined NPI effectiveness in reducing disease transmissivity deriving from the modified model fitting with observed daily reported cases. Results are shown using a violin plot and the box plot inside each violin display median and interquartile range, Figure S6, Implementing a daily quota for inbound travelers and community NPIs to contain the importation risk during the 120-day simulation period (90% successful using contact tracing for individuals). The number of daily reported cases are estimated under scenarios with different daily quotas (no quota, 50% of historical inflow, and 10,000 population) and community NPIs (no, moderate, and strong). The color shaded areas are the 95% CI of estimated daily reported cases, Table S1: Description of combined community NPIs implemented in designated scenarios, Table S2: Implementing a daily quota for inbound travelers and community NPIs with contact tracing (90% successful for individuals) in containing importation risk for reopening borders.
