# Supplementary material for: Reopening International Borders without Quarantine: Contact Tracing Integrated Policy against COVID-19
Source: Int J Environ Res Public Health. 2021 Jul 14;18(14):7494. doi: 10.3390/ijerph18147494 (PMC8303901; doi:10.3390/ijerph18147494)
Supplement: Supplementary file 1 [file ijerph-18-07494-s001.zip › ijerph-1290525-supplementary.pdf]

Supplemental material

Table S1 Description of combined community NPIs implemented in designated scenarios

Level	Description
Moderate	School arrangement (Mild), Suspension of large events (Moderate), Public facility arrangement (Mild), Restricting social gathering (Moderate), Voluntary check-in system, Compulsory testing
Strong	School arrangement (Moderate), Suspension of large events (Strict), Public facility arrangement (Moderate), Restricting social gathering (Strict), Voluntary check-in system, Compulsory testing

Table S2 Implementing a daily quota for inbound travelers and community NPIs with contact tracing (90% successful for individuals) in containing importation risk for reopening borders.

Daily quota	Community NPIs	Cumulative cases during the first 60 days (95%CI)	Cumulative cases during the first 60 days (95%CI)	Reduction rate of cases (%)
		No contact tracing	Contact tracing	
No	No	129638(32387-229935)	20723(12288-28314)	84.01
	Moderate	18460(15548-21643)	10983(9913-12042)	40.50
	Strong	15588(13907-17489)	10705(9683-11670)	31.32
50% of historical inflow	No	57278(17796-98606)	10571(5496-14700)	81.54
	Moderate	8772(7613-10354)	5473(4792-5978)	37.60
	Strong	7762(6739-8642)	5208(4711-5859)	32.90
10,000	No	6700(2166-10102)	1113(306-2687)	83.38
	Moderate	960(725-1210)	655(469-733)	31.77
	Strong	859(647-1027)	634(457-716)	26.19

Note: The reduction rate refers to the percentage of cases that could be reduced if the digital contact tracing is applied, compared with the identical scenario without contact tracing.

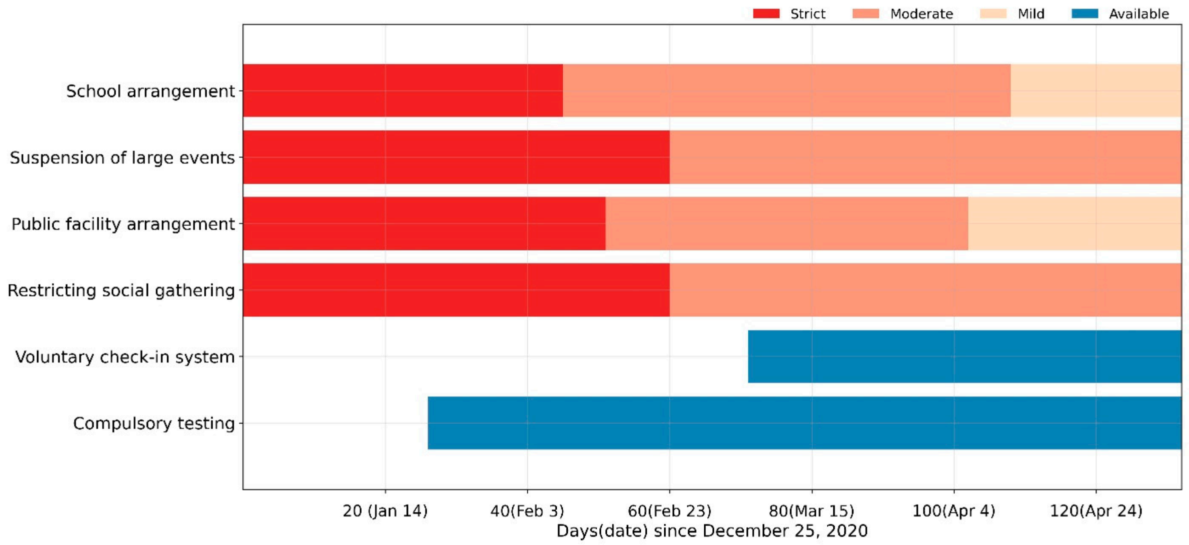

Figure S1 Gantt chart for the implementation period of NPIs. Different colors represent levels of strictness in terms of the implemented NPIs.

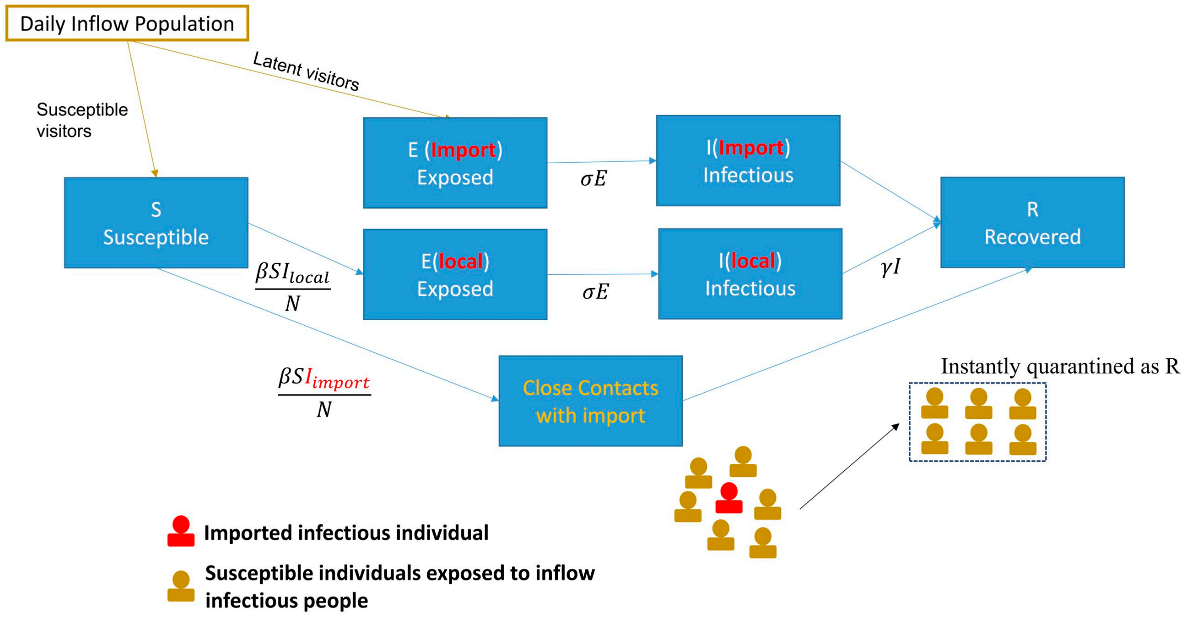

Figure S2 Schematic to explain this rationale how digital contact tracing help to contain the importation risks. In this illustration, we assume that close contacts exposed to the inflow infectious individual are ideally (100%) self-isolated or mandatory quarantined.

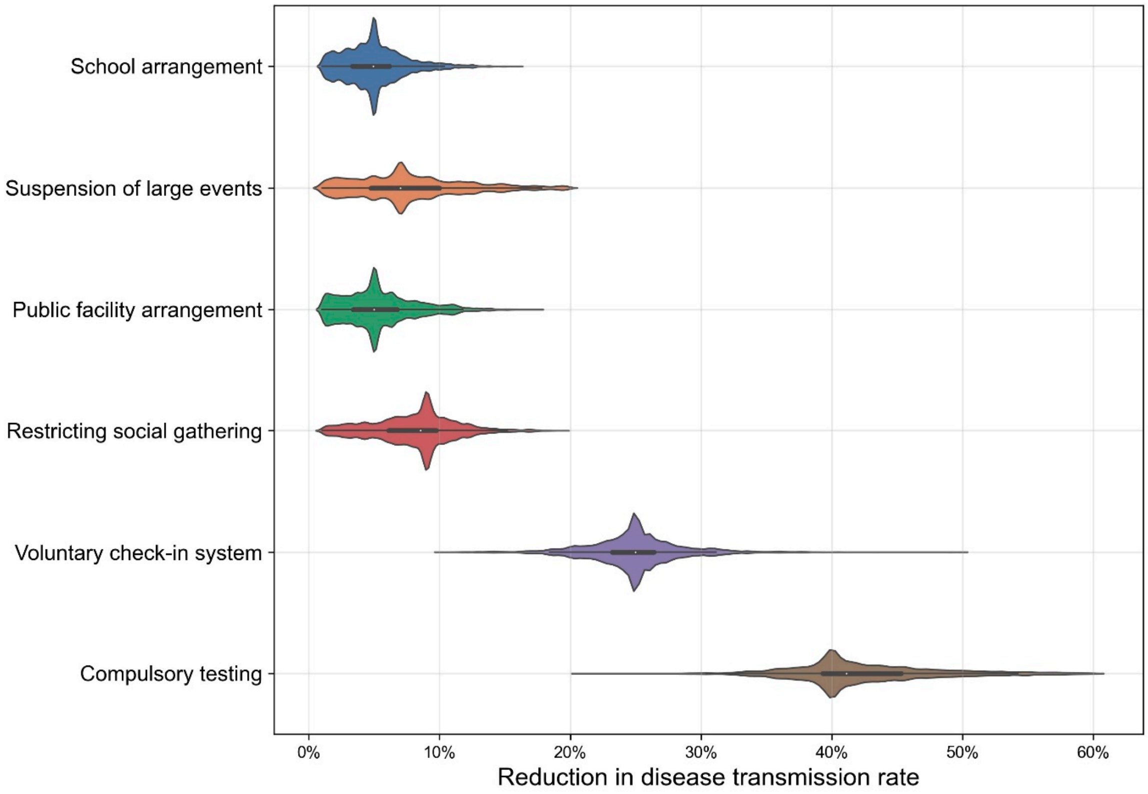

Figure S3 Individual NPI effectiveness in reducing disease transmissivity deriving from the modified model fitting with observed daily reported cases. Results are shown using a violin plot and the box plot inside each violin display median and interquartile range.

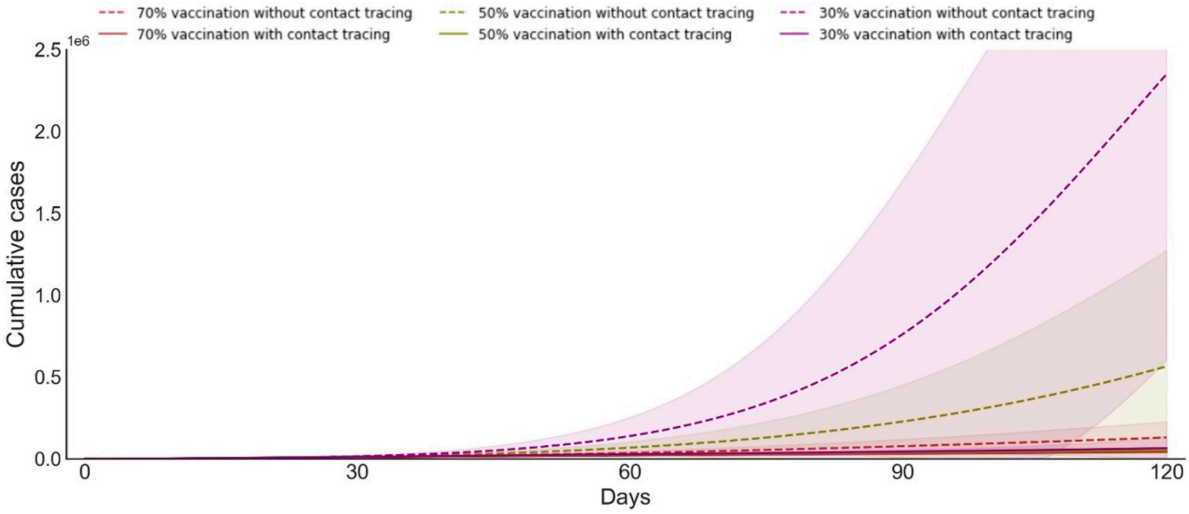

Figure S4 Estimated cumulative daily cases under three different vaccination coverage. The color shaded area shows the 95% CI of estimated cumulative cases regarding corresponding scenarios. Solid and dotted lines represent the scenarios with and without contact tracing, respectively. Different colors represent scenarios with 70%, 50%, and 30% vaccination coverage.

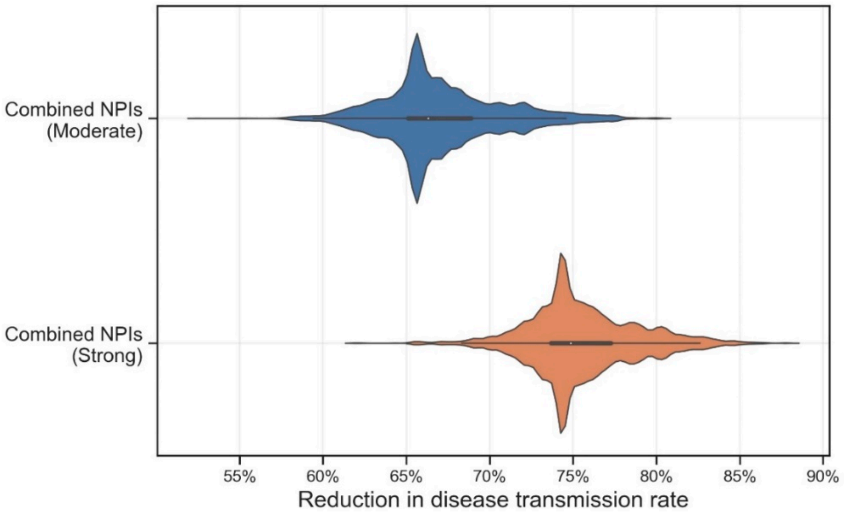

Figure S5 Combined NPI effectiveness in reducing disease transmissivity deriving from the modified model fitting with observed daily reported cases. Results are shown using a violin plot and the box plot inside each violin display median and interquartile range.

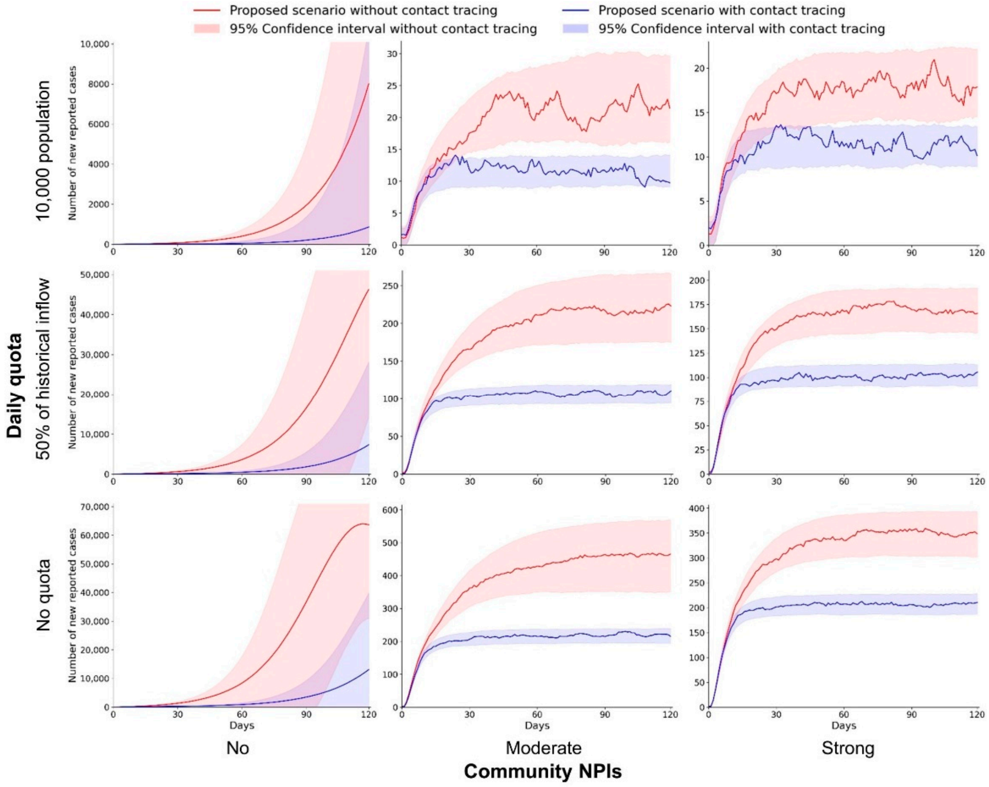

Figure S6 Implementing a daily quota for inbound travelers and community NPIs to contain the importation risk during the 120-day simulation period (90% successful using contact tracing for individuals). The number of daily reported cases are estimated under scenarios with different daily quotas (no quota, 50% of historical inflow, and 10,000 population) and community NPIs (no, moderate, and strong). The color shaded areas are the 95% CI of estimated daily reported cases.
